# Efeitos dos Marca-passos nas Funções Ventriculares e Variáveis Precoces Associadas à Cardiomiopatia Induzida

**DOI:** 10.36660/abc.20250566

**Published:** 2026-07-31

**Authors:** Sanjeev Kumar, Chandra Bhan Meena, Navida Babbar, Anil Bhandari, Dhananjay Singh Shekhawat, Ankita Meel

**Affiliations:** 1 SMS Medical College Jaipur Índia SMS Medical College, Jaipur – Índia

**Keywords:** Marca-Passo Artificial, Insuficiência Cardíaca, Ecocardiografia Tridimensional, Artificial, Cardiomiopatias

## Abstract

**Fundamento:**

A estimulação do ventrículo direito pode alterar a mecânica ventricular e levar à disfunção ventricular, mesmo em pacientes com fração de ejeção do ventrículo esquerdo (FEVE) basal preservada e corações estruturalmente normais.

**Objetivo:**

Este estudo examina os efeitos hemodinâmicos da estimulação do ventrículo direito sobre a função e o volume do ventrículo esquerdo e identifica variáveis precoces associadas à disfunção ventricular esquerda induzida por marca-passo e à cardiomiopatia em pacientes com marca-passos de dupla câmara no ventrículo direito (VD).

**Métodos:**

O estudo incluiu pacientes submetidos a implante de marca-passo de dupla câmara no VD sem qualquer anormalidade estrutural do coração. Dados sobre as funções biventriculares, volumes do VE e *strain* longitudinal global (SLG) foram coletados por meio de ecocardiografia bidimensional, com rastreamento de *speckle* e ecocardiografia tridimensional. Variáveis contínuas pareadas foram comparadas utilizando testes bicaudais com nível de significância de 0,05.

**Resultado:**

Dos 75 pacientes, 20 apresentaram disfunção sistólica do VE, 15 desenvolveram disfunção ventricular induzida por marca-passo (PIVD) e cinco desenvolveram cardiomiopatia induzida por marca-passo (PICM). Em comparação com o grupo controle, os pacientes com PICM apresentaram SLG mais baixo (17,02 vs. 19,27; p<0,05), maior duração do QRS (139,57 vs. 102,75; p < 0,05) e maior porcentagem de estimulação ventricular (EV%; 94,32 vs. 84,03; p = 0,06).

**Conclusão:**

A estimulação do VD resultou em um declínio substancial da função sistólica do VE a curto e longo prazo. Concomitantemente, as funções do VD declinaram no longo prazo (seis meses). A diminuição do SLG aos sete dias, um complexo QRS pré-implante mais amplo e uma elevação de EV% são indicadores precoces de PIVD e PICM. O monitoramento rotineiro desses indicadores pode ser benéfico para a intervenção oportuna, caso a caso.

## Graphical abstract

**Figura Central:**
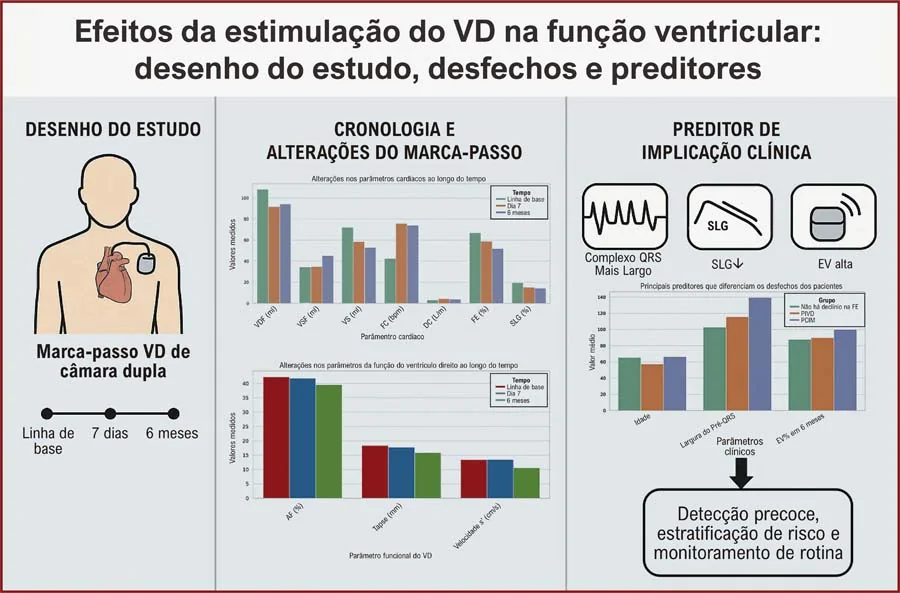
: Efeitos dos Marca-passos nas Funções Ventriculares e Variáveis Precoces Associadas à Cardiomiopatia Induzida

Ilustrando o protocolo do estudo, as alterações temporais na função ventricular e as variáveis associadas a desfechos adversos (exploratórios) com estimulação ventricular direita bicameral.

## Introdução

Pacientes com fração de ejeção do ventrículo esquerdo (FEVE) basal preservada e corações estruturalmente normais representam uma população crescente que necessita de implante de marca-passo permanente.^1-3^ A estimulação cardíaca, uma terapia bem estabelecida para o tratamento da insuficiência cardíaca e arritmias, desempenha um papel crucial em pacientes com bradicardia ou bloqueio atrioventricular, nos quais os marca-passos são essenciais para a manutenção de um ritmo cardíaco adequado.^4^ Ao longo das décadas, a tecnologia de estimulação cardíaca evoluiu de marca-passos de câmara única para dispositivos de câmara dupla e, posteriormente, para marca-passos biventriculares na terapia de ressincronização cardíaca (TRC), que melhora o débito cardíaco em pacientes com função do VE reduzida.^5,6^ No entanto, a TRC não é indicada rotineiramente em indivíduos com FEVE preservada, e seu alto custo e complexidades técnicas limitam sua aplicação mais ampla.^7^

Em pacientes que necessitam de implante de marca-passo permanente, que apresentam FE basal preservada e corações estruturalmente normais, a estimulação do ventrículo direito (VD) permanece sendo a estratégia mais comumente empregada.^8^ A estimulação prolongada do VD induz dissincronia ventricular, que interrompe a função cardíaca normal, levando à disfunção ventricular induzida por marca-passo (PIVD) e cardiomiopatia induzida por marca-passo (PCIM), piorando a insuficiência cardíaca e aumentando a mortalidade.^9,10^ A identificação precoce de pacientes em risco é crucial para prevenir complicações graves.^11,12^

Este estudo tem como objetivo investigar os efeitos hemodinâmicos da estimulação do VD sobre os volumes e funções do VE, avaliar a função do VD e identificar variáveis precoces associadas às PIVD e PCIM em pacientes com marca-passo bicameral no VD. Além disso, este estudo visa fornecer insights que possam orientar a tomada de decisões clínicas, melhorar o manejo do paciente e, em última análise, reduzir o impacto da insuficiência cardíaca em pacientes dependentes de estimulação do VD.

## Pacientes e Métodos

### Desenho e aprovação do estudo

O estudo foi aprovado pelo nosso comitê de ética institucional em 04/04/2023, e todos os pacientes participantes deram seu consentimento livre e esclarecido por escrito. O recrutamento ocorreu entre março de 2022 e março de 2023, quando 200 pacientes consecutivos procuraram nosso hospital para implante de marca-passo.

### Seleção de pacientes

Pacientes com FE < 50%, valvopatia significativa, doença arterial coronariana, janelas ecocardiográficas inadequadas, fibrilação atrial persistente com resposta ventricular tardia, outras arritmias que potencialmente afetem a função do VE, qualquer condição debilitante ou câncer (com expectativa de sobrevida inferior a um ano), implante prévio de dispositivos e cirurgia cardíaca recente (nos últimos três meses) foram excluídos. Do grupo inicial, apenas 75 pacientes preencheram os critérios e, portanto, foram incluídos no estudo. A população do estudo foi composta por pacientes com FEVE basal preservada (≥ 50%) e corações estruturalmente normais, conforme confirmado por ecocardiografia. Os pacientes não foram avaliados quanto ao diagnóstico clínico de insuficiência cardíaca e nenhuma classificação de insuficiência cardíaca foi utilizada como critério de inclusão.

### Implantação e programação de marca-passo

Todos os pacientes deste estudo foram submetidos a implante de marca-passo ventricular direito de dupla câmara. Os procedimentos de implante foram realizados de acordo com a preferência do operador. Em 65 pacientes, o septo interventricular (SVD) foi escolhido como local de implante do eletrodo VD. Os dez pacientes restantes foram submetidos a estimulação apical do VD (AVD). O local de estimulação foi confirmado por avaliação do eixo eletrocardiográfico (ECG) após o implante. Um modo de modulação de frequência de dupla câmara foi configurado para todos os pacientes. O modo responsivo à frequência foi empregado para melhorar a qualidade de vida e a capacidade de exercício dos pacientes. Os limites inferiores de frequência foram definidos em 60 batimentos por minuto e os limites superiores em 130 batimentos por minuto. O atraso atrioventricular em repouso para os sinais estimulados/detectados foi definido em 200/150 ms, com otimização do atraso atrioventricular dinâmico visando maximizar a contribuição da contração atrial ao volume sistólico. Estratégias para reduzir a estimulação foram implementadas.^13^

### Coleta de dados

Os dados foram coletados três vezes: antes do implante do marca-passo, sete dias e seis meses após o implante. As alterações nas configurações de estimulação foram verificadas e a EV% foi obtida por meio da análise do dispositivo. Durante as consultas de acompanhamento, as avaliações incluíram ecocardiografia 2D e 3D, bem como avaliação do *strain* longitudinal global (SLG). A FE% foi utilizada para classificar os pacientes com PIVD ou PCIM. Uma redução superior a 10%, mantendo a FE > 50%, é definida como PIVD, enquanto a PCIM é caracterizada por uma redução superior a 10%, resultando em FE ≤ 50%.^14^

### Eletrocardiograma

Antes da estimulação cardíaca, foi realizado um ECG padrão de 12 derivações. Os dados do ECG, incluindo frequência cardíaca, duração e eixo do complexo QRS nativo, grau de bloqueio cardíaco e presença de morfologia semelhante a bloqueio de ramo esquerdo (BRE) ou bloqueio de ramo direito (BRD), foram coletados e documentados. A determinação de BRE e BRD seguiu critérios estabelecidos. Posteriormente, todos os parâmetros do ECG foram reavaliados e documentados sete dias e seis meses após a estimulação cardíaca.

### Ecocardiografia bidimensional

Para excluir indivíduos com doença cardíaca coronária, doença cardíaca valvular significativa, ou fração de ejeção reduzida, foi realizado um exame ecocardiográfico bidimensional completo.

### Parâmetros do Ventrículo Direito

A função sistólica do ventrículo direito foi avaliada utilizando a variação fracional da área (FAC), a excursão sistólica do plano anular tricúspide (TAPSE) e a velocidade sistólica derivada do Doppler tecidual (S’) obtida na vista apical de quatro câmaras, de acordo com as recomendações ecocardiográficas atuais.^15^ A variação fracional da área foi calculada como [(área diastólica final do VD − área sistólica final do VD) / área diastólica final do VD] × 100. A imagem Doppler tecidual foi realizada posicionando o volume de amostra no anel tricúspide lateral para obter a velocidade sistólica de pico (S’).

#### Medição SLG

As imagens apicais (apicais quatro, dois e três) foram adquiridas em 2D com sincronização respiratória para o cálculo do GLS utilizando métodos padrão da indústria. Para garantir imagens nítidas e minimizar a extração de imagens, os pacientes foram instruídos a prender a respiração durante a aquisição das imagens. De acordo com os cálculos do GLS, o GLS central foi determinado automaticamente para cada uma das três vistas apicais (Figura 1).

**Figura 1:**
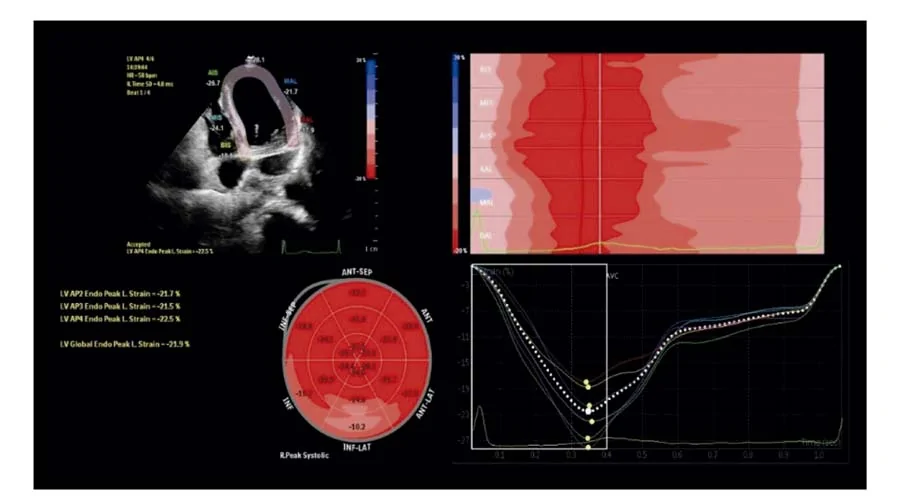
– Imagens representativas para medições de SLG. O SLG foi calculado a partir de três vistas apicais padrão no conjunto de dados ecocardiográficos 2D.

#### Ecocardiografia 3D

Para a ecocardiografia 3D, utilizamos o sistema de ecocardiografia Philips Medical IE33 com uma sonda transdutora X5-1. Obtivemos uma pirâmide de 30° x 30° com mais de 4 a 6 ciclos cardíacos alternados e sincronizados no ventrículo esquerdo. Garantimos tamanho de pacote ideal, taxa de quadros adequada e registro completo do ciclo cardiovascular (Figura 2).

**Figura 2:**
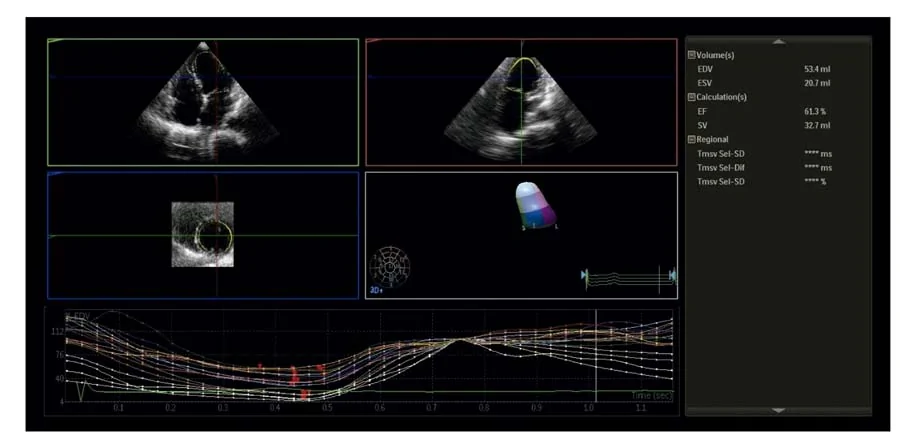
– Esta figura apresenta cortes bidimensionais de um conjunto de dados ecocardiográficos 3D do miocárdio do ventrículo esquerdo, usados para calcular parâmetros cardíacos importantes: VDF, VSF, VS, DC e FE.

## Parâmetros do ventrículo esquerdo

O programa delimitou as bordas do ventrículo esquerdo, permitindo que os usuários fizessem ajustes para maior precisão, conforme necessário. Os parâmetros do VE: débito cardíaco (DC), volume sistólico (VS), volume diastólico final (VDF), volume sistólico final (VSF) e FE foram calculados automaticamente após o desenho das bordas.

## Análise estatística

Um grupo independente de gerenciamento de dados foi encarregado de manter e analisar o banco de dados. Para avaliar a distribuição dos dados das variáveis contínuas, foram calculados a assimetria e a curtose padronizadas. Os dados com distribuição normal foram representados como média ± desvio padrão (DP). As variáveis categóricas foram expressas como frequências absolutas e percentagens. A significância estatística foi considerada para todas as comparações entre grupos com p < 0,05. Comparações pareadas bicaudais entre os pontos de tempo foram realizadas utilizando o teste-t de Student pareado. As comparações entre os três grupos de desfecho funcional do VE (sem declínio, PIVD e PICM) foram realizadas utilizando análise de variância (ANOVA) de uma via para variáveis contínuas e o teste χ^2^ ou o teste exato de Fisher para variáveis categóricas, conforme apropriado. Quando a ANOVA geral foi estatisticamente significativa (p < 0,05), as comparações entre os grupos foram realizadas utilizando o teste post-hoc de Tukey (HSD) para identificar quais grupos específicos diferiram significativamente, controlando a taxa de erro familiar. Foram calculadas estimativas de efeito com intervalos de confiança de 95% para parâmetros funcionais chave.

## Resultados

### Características basais e fatores de risco dos participantes do estudo

Um total de 75 pacientes foram incluídos e completaram as observações definidas 6 meses após os testes subsequentes. A média de idade dos pacientes era de 65 anos, variando entre 12 e 97 anos. Vinte pacientes (26,7%) tinham diabetes e 32 pacientes (42,7%) tinham hipertensão. Além disso, 22 pacientes (29,3%) apresentavam BRE ou BRD pré-existente (Tabela 1). O ecocardiograma confirmou a normalidade estrutural do coração em todos os pacientes. Uma ilustração central resume o desenho do estudo, a cronologia das alterações relacionadas ao marca-passo e as principais variáveis associadas aos desfechos da função ventricular em pacientes com marca-passos ventriculares de dupla câmara no VD.

**Tabela 1:** – Resumo das características dos pacientes

Fatores	Número	%
**Idade (Média ± DP)**	65,30 ± 10,09	
**Sexo**		
Masc.	40	53,33
Fem.	35	46,67
**DM**	20	26,66
**HAS**	32	42,67

DM: diabetes mellitus; HAS: hipertensão arterial sistêmica.

### Resposta ventricular à estimulação

Observamos uma redução significativa nos volumes e funções do VE após a estimulação (Tabela 2). Especificamente, o VDF e o VS apresentaram uma redução notável já sete dias após a estimulação. Além disso, o VSF aumentou seis meses após a estimulação, em comparação com os valores basais. A FE e o SLG demonstraram uma redução acentuada no sétimo dia, com essas reduções continuando progressivamente até o período de seis meses após a estimulação, em comparação com as medidas basais.

**Tabela 2:** – Comparação de diferentes parâmetros em grupos de estimulação bicameral, incluindo todos os parâmetros medidos nos três intervalos de tempo (pré-estimulação, 1 semana e 6 meses pós-estimulação)

Parâmetros	Pré-estimulação	Pós-estimulação no 7º dia	Pós-estimulação aos 6 meses	Valor de p (Linha de base vs. 6 meses)	Variação média (Linha de base até 6 meses): IC de 95%
VDF (ml)	108,25 ± 12,49	91,65 ± 7,93*	94,26 ± 12,36^$^	<0,001	–14,0 (IC 95% –16,5 a –11,6)
VSF (ml)	34,38 ± 6,43	34,92 ± 6,18	45,32 ± 6,20^$#^	<0,001	+10,9 (IC 95% +10,5 a +11,3)
VS (ml)	72,28 ± 10,20	58,48 ± 8,32*	53,01 ± 5,11^$#^	<0,001	–19,3 (IC 95% –21,1 a –17,5)
FC (bpm)	42,44 ± 5,90	75,75 ± 5,69*	73,89 ± 4,86^$^	<0,001	+31,5 (IC 95% +29,8 a +33,2)
DC (L/m)	3,02 ± 0,52	4,42 ± 0,52*	3,90 ± 0,52^$#^	<0,001	+0,88 (IC 95% +0,80 a +0,96)
FE (%)	66,84 ± 12,19	58,84 ± 17,18*	52,04 ± 14,18^$#^	<0,001	–14,80 (–19,07 a –10,53)
SLG absoluto (%)	19,54 ± 1,98	15,22 ± 1,97*	14,33 ± 1,93^$^	<0,001	–5,20 (IC 95% –5,84 a –4,58)

Os valores são apresentados como média ± desvio padrão (DP). Os valores de p representam comparações do teste-t de Student pareado entre as medidas basais e as medidas após seis meses. *p < 0,001 em comparação com o valor basal (valor basal em comparação com o dia 7); p < 0,001 em comparação com o valor basal (valor basal em comparação com 6 meses); ^#^p < 0,001 vs. dia 7 (dia 7 vs. 6 meses). VDF: volume diastólico final; VSF: volume sistólico final; VS: volume sistólico; FC: frequência cardíaca; DC: débito cardíaco; FE: fração de ejeção; SLG: strain longitudinal global.

As funções do VD, AF, TAPSE e velocidade S’ foram inicialmente mantidas sete dias após a estimulação. No entanto, esses parâmetros apresentaram um declínio significativo seis meses após a estimulação, em comparação com seus respectivos valores basais (Tabela 3). Esses dados destacam os impactos da estimulação nas funções do VE e do VD ao longo do tempo.

**Tabela 3:** – Comparação de diferentes parâmetros em grupos de estimulação bicameral, incluindo todos os parâmetros medidos do ventrículo direito nos três intervalos de tempo (pré-estimulação, 1 semana e 6 meses pós-estimulação)

Parâmetros	Pré-estimulação	Pós-estimulação no 7º dia	Pós-estimulação aos 6 meses	Valor de p (Linha de base vs. 6 meses)	Variação média (Linha de base até 6 meses): IC de 95%
AF (%)	42,28 ±2,16	41,82 ± 2,39	39,55 ± 3,28^$#^	<0,001	–1,95 (–3,85 a –0,06)
TAPSE (mm)	18,39 ± 2,20	17,77 ± 2,31	15,91 ± 2,98^$#^	<0,001	–3,21 (–5,13 a –1,30)
Velocidade S (cm/s)	13,43 ± 1,03	13,51 ± 1,30	10,61 ± 1,44^$#^	<0,001	–2,38 (–3,23 a –1,53)

Os valores são apresentados como média ± desvio padrão (DP). Os valores de p representam comparações do teste t de Student pareado entre as medidas basais e as medidas após seis meses. *p < 0,001 em comparação com o valor basal (valor basal em comparação com o dia 7); p < 0,001 em comparação com o valor basal (valor basal em comparação com 6 meses); ^#^p < 0,001 vs. dia 7 (dia 7 vs. 6 meses). AF: área fracional; TAPSE: excursão sistólica do plano anular tricúspide.

### Estratificação de risco para cardiomiopatia induzida por marca-passo

Sete dias após o implante do marca-passo, 55 pacientes apresentaram redução da FE inferior a 10% (grupo controle), enquanto 20 apresentaram redução da FE ≥ 10% em comparação com a FE pré-implante (Tabela 4). Uma análise de subgrupo desses 20 pacientes identificou 15 pacientes com PIVD (FE ≥ 50%) e cinco pacientes com PICM (FE < 50%). Aos seis meses, a FE média nos grupos PIVD e PICM diminuiu 23,7% e 41,2%, respectivamente.

**Tabela 4:** – Comparação dos fatores de risco nos grupos de estudo: (SVD - estimulação septal do VD, AVD - estimulação apical do VD)

Fator predisponente	Sem declínio na FEVE (55)	Diminuição da FEVE (fração de ejeção do ventrículo esquerdo)		Valor de p
		PIVD (15)	PICMP (5)	
**Idade**	65,30 ± 10,09	57,6 ± 11,72	66,4 ± 11,85	0,03
**Sexo**	n (%)	n (%)	n (%)	0,81
Masc	30 (54,55)	7 (46,67)	3 (60)	
Fem	25 (45,55)	8 (53,33)	2 (40)	
**DM**	14 (25,45)	3 (20)	3 (60)	0,06
**HAS**	24 (43,63)	5 (33,33)	3 (60)	0,07
**Site principal**				0,67
AVD (10)	7 (12,7)	2 (13,33)	1 (20)	
SVD (65)	48 (87,27)	13 (87,67)	4 (80)	
**Bloqueio pré-existente**				0,06
Sem bloqueio	44 (80)	9 (60)	0 (0,0)	
BRD	8 (14,55)	4 (26,67)	4 (80)	
BRE	3 (5,45)	2 (13,33)	1 (20)	
**Eixo QRS**				0,78
N	38 (69,1)	11 (73,4)	1 (20)	
Esquerda	5 (9.1)	2 (13.3)	2 (40)	
Direita	12 (21,8)	2 (13.3)	2 (40)	
**Largura do complexo QRS nativo pré-implante (ms)**				
Pré-estimulação	102,75 ± 15,37	115,61 ± 19,48	139,57 ± 19,77	<0,05
Pós-estimulação	147,24 ± 13,25	153,11 ± 12,83	157,57 ± 17,35	0,74
**EV%**				
7 dias	84,03 ± 14,64	82,53 ± 9,67	94,32 ± 5,1	0,06
6 meses	87,68 ± 11,34	89,79 ± 8,79	100,00 ± 0,0	0,021
**Limiar de estimulação**				
7 dias	0,56 ± 0,11	0,54 ± 0,11	0,49 ± 0,1	0,37
6 meses	0,84 ± 0,15	0,87 ± 0,12	0,82 ± 0,32	0,92

FEVE: fração de ejeção do ventrículo esquerdo; PIVD: disfunção ventricular induzida por marca-passo; PICM: cardiomiopatia induzida por marca-passo. AVD: apical do ventrículo direito; SVD: septo interventricular; BRE: bloqueio de ramo esquerdo; BRD: bloqueio de ramo direito; EV: estimulação ventricular.

Os grupos PIVD e PICM apresentaram uma redução no SLG sete dias após o implante em comparação com o SLG pré-implante (Figura 3a) e apresentaram intervalos QRS pré-implante mais amplos do que o grupo controle (Figura 3b). O grupo PICM também apresentou um SLG pré-implante baixo (Figura 3a) e um EV% mais elevado (Tabela 4) em comparação com os grupos PIVD e controle. Quando a ANOVA de uma via foi estatisticamente significativa (p < 0,05), o teste post-hoc de Tukey (HSD) foi aplicado para identificar diferenças específicas entre os grupos. Os resultados completos das comparações pareadas são apresentados na Tabela 5. A duração do complexo QRS pré-implante diferiu significativamente entre os três grupos (p < 0,05), com o teste HSD de Tukey confirmando um aumento gradual em todas as comparações pareadas: Sem Declínio vs. PIVD (p = 0,025), Sem Declínio vs. PICM (p < 0,001) e PIVD vs. PICM (p = 0,017). A EV% aos seis meses diferiu significativamente entre os grupos (p = 0,021), impulsionada principalmente por uma EV% maior no grupo PICM em comparação com o grupo Sem Declínio (p = 0,039). A idade apresentou uma diferença geral significativa (p = 0,030), com uma média de idade menor no grupo PIVD em comparação com o grupo Sem Declínio (p = 0,038). Para todas as demais variáveis, a ANOVA geral não foi significativa (p ≥ 0,05) e, portanto, os testes post-hoc não foram realizados (Tabela 5).

**Figura 3:**
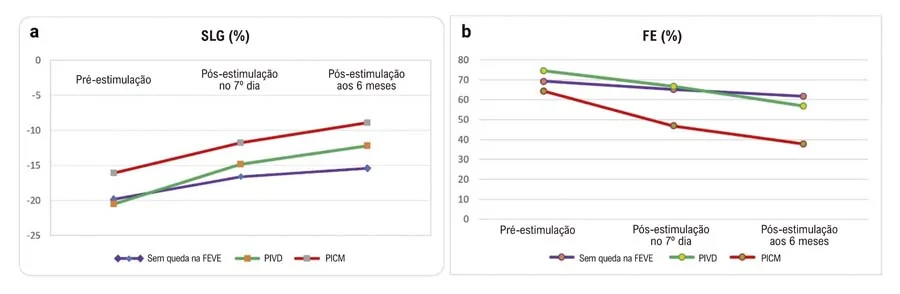
– Variáveis precoces associadas à PIVD e PICM (a) Um complexo QRS pré-implante mais amplo e (b) um SLG pós-implante baixo sugerem um risco maior de disfunção cardíaca.

**Tabela 5:** – Comparações pareadas post-hoc de Tukey (HSD) após ANOVA de uma via para variáveis de estratificação de risco (Tabela 4)

Variável	Sem declínio vs PIVD	Sem declínio vs. PICM	PIVD vs PICM
Idade	p = 0,038*	p = 0,973 (ns)	p = 0,245 (ns)
Largura do complexo QRS nativo pré-implante (ms)	p = 0,025*	p < 0,001***	p = 0,017*
EV% aos 6 meses (%)	p = 0,772 (ns)	p = 0,039*	p = 0,154 (ns)

Variáveis com ANOVA não significativa (p ≥ 0,05): pós-teste não aplicável. ND: sem declínio; PIVD: disfunção ventricular induzida por marca-passo; PICM: cardiomiopatia induzida por marca-passo; ns: não significativo; N/A, não aplicável (teste post-hoc não realizado quando ANOVA geral p ≥ 0,05). Significância: * p < 0,05; ** p < 0,01; *** p < 0,001.

## Discussão

Neste estudo exploratório, a disfunção sistólica do VE induzida por estimulação foi mais prevalente, manifestando-se em até seis meses após o implante. Um complexo QRS pré-implante mais amplo, um SLG pós-implante mais baixo (uma alteração precoce observada em sete dias) e uma maior porcentagem de estimulação foram associados ao desenvolvimento de PICM e PIVD.

Observamos impactos adversos de curto e longo prazo no volume e nas funções do VE. Em sete dias, o VS foi significativamente reduzido em associação com uma queda no VDF. Isso pode estar relacionado à estimulação em frequências cardíacas mais elevadas, encurtando a duração do cLiclo cardíaco, particularmente a diástole, e reduzindo o tempo para o enchimento ventricular. Essa diminuição no VDF reduz o estiramento miocárdico, consistente com uma queda no VS (lei de Frank-Starling).^16^ Yu et al. investigaram os efeitos agudos da estimulação ventricular direita em 35 pacientes com síndrome do nó sinusal, observando uma redução no VDF e na FE do VE.^17^ A redução da FE pode estar associada à remodelação da estrutura do VE induzida pela estimulação, que alterou os volumes do VE, principalmente aumentando o VSF.^18^ No segundo acompanhamento, também observamos uma queda adicional na FE e um aumento no VSF, o que pode estar relacionado à remodelação da estrutura do VE induzida pela estimulação.

Identificamos 20 (27%) pacientes com redução ≥10% na FE, posteriormente categorizados em pacientes com PIVD (20%) ou PICM (7%). É importante notar que o número de casos de PICM foi pequeno (n=5), o que limita conclusões definitivas e impede o ajuste multivariável. A incidência de PICM em nosso estudo está de acordo com relatos anteriores de 5-27%, com a variação dependendo dos critérios de definição de PICM e da duração do acompanhamento.^14^ Lee et al. encontraram uma incidência de PICM de 20,5% após um período médio de acompanhamento de 15,6 anos, enquanto Dawood et al. relataram uma taxa de incidência de 12% com um acompanhamento de seis meses.^19,20^

Observou-se um SLG pré-implante absoluto mais baixo em pacientes que posteriormente desenvolveram PICM. Uma redução significativa no SLG absoluto foi observada em sete dias em pacientes com PIVD ou PICM. Um número crescente de pesquisas destaca a associação negativa entre estimulação cardíaca e contração miocárdica. Delgado et al. relataram os efeitos da estimulação do átrio direito (AVD) em 25 indivíduos com corações estruturalmente normais, encontrando maior contração dissincrônica do VE e comprometimento do encurtamento longitudinal e da torção do VE durante a estimulação do AVD.^21^ Babu et al. acompanharam 36 pacientes com FE acima de 45% por seis meses após o implante de marca-passo permanente, observando uma redução significativa no SLG em 63,9% dos pacientes em seis meses, com uma redução notável na deformação longitudinal geral em sete pacientes 24 horas após o implante.^22^ Estudos recentes indicam que pacientes com SLG mais baixo podem estar em risco de desenvolver PICM, identificando o pico de SLG do VE mais baixo como um indicador independente de dissincronia do VE.^23^

O grupo PICM apresentou um complexo QRS pré-implante mais amplo e uma EV% mais alta em ambos os acompanhamentos. Outro estudo constatou que sexo masculino, FE basal mais baixa e um complexo QRS nativo amplo foram variáveis associadas à PICM.^24^ Kim et al. também associaram a PICM a um eixo QRS pós-estimulação anormal, o que nosso estudo não pôde corroborar devido ao pequeno tamanho da amostra.^25^

Observamos uma redução significativa no TAPSE, na velocidade S’ e na AF no segundo acompanhamento, indicando disfunção do VD. Sinkar et al. avaliaram as dimensões e a função do VD em 60 pacientes seis meses após o implante de marca-passo, constatando um declínio nos parâmetros de função do VD.^26^ Porapakkham et al. analisaram os fatores que afetam a disfunção do VD em 96 pacientes, observando que os fatores que afetam a função do VE, como o local do eletrodo de estimulação, o modo de estimulação e a EV%, não impactaram significativamente a função do VD.^27^

Nosso estudo foi limitado devido ao pequeno tamanho da amostra e ao curto período de acompanhamento, embora tenha sido estatisticamente significativo. Um acompanhamento mais longo permitiria prever uma maior incidência de disfunção induzida por estimulação. Além disso, 87% dos pacientes em nosso estudo foram submetidos à estimulação do SVD, com apenas 10 (13%) pacientes submetidos à estimulação do AVD. Devido a isso, nosso estudo é limitado na análise do papel do local de implante do eletrodo no VD sobre as funções ventriculares e a cardiomiopatia. Alguns estudos mostraram que a estimulação do SVD apresenta melhores resultados clínicos a curto e longo prazo. Karpawich et al. compararam locais de estimulação apical e médio-septal em cães, constatando que a estimulação septal preveniu a remodelação durante um período de acompanhamento de quatro meses.^28^ Eles encontraram morfologia mitocondrial preservada, sem calcificação ou alterações degenerativas no grupo com estimulação septal.

Tse et al. relataram que pacientes com estimulação do AVD apresentaram duração média do QRS significativamente maior, FE mais baixa e maior incidência de defeitos de perfusão miocárdica e anormalidades regionais do que pacientes com estimulação do SVD.^29^ Eles concluíram ainda que a preservação da ativação ventricular síncrona durante a estimulação do SVD atenua os efeitos deletérios a longo prazo da estimulação do AVD sobre a perfusão e a função miocárdicas.^16^ Entretanto, Ng et al. demonstraram, em um estudo de longo prazo, que a estimulação septal resultou em pior dissincronia do VE e em uma deformação circunferencial mais comprometida do que a estimulação apical.^30^

Diversas fontes potenciais de viés devem ser consideradas na interpretação dos resultados. As medidas de deformação e da função do ventrículo direito são inerentemente dependentes do operador e influenciadas pela qualidade da imagem e pelos algoritmos de rastreamento. Embora tenham sido utilizados protocolos de aquisição padronizados e operadores experientes, a variabilidade interobservador não pode ser completamente excluída e pode ter afetado a precisão dos índices de *strain* e da função do VD. Apesar de suas contribuições inéditas, o estudo apresenta algumas limitações. Embora nenhum cálculo a priori do tamanho da amostra tenha sido realizado, uma análise de poder post-hoc baseada na redução observada da FE de 14,8 pontos percentuais (DP ~13,0) em 75 pacientes demonstrou poder >99% com α = 0,05, e a redução observada do SLG de 5,2% (DP ~2,0) também apresentou poder >99%. Esses achados sugerem poder estatístico adequado para os desfechos ecocardiográficos primários; no entanto, o pequeno número de casos de PICM limitou as análises de subgrupos e a modelagem multivariável. Devido à baixa contagem de eventos de PICM, o ajuste multivariável não foi possível e a presença de fatores de confusão residuais não pode ser descartada. As medições de *strain* e ecocardiografia 3D dependem do operador e são suscetíveis a variações na qualidade da imagem.

São necessários mais estudos com acompanhamento prolongado para elucidar os efeitos a longo prazo da estimulação cardíaca na função do VE, visto que uma observação mais longa pode revelar mais casos de disfunção. Tamanhos de amostra maiores aumentarão o poder estatístico para conclusões definitivas e análises de subgrupos, particularmente em relação aos locais de estimulação. Considerando os diferentes resultados da estimulação do AVD e do SVD, estudos comparativos com avaliações padronizadas de dissincronia e perfusão são essenciais. Investigar a fibrose miocárdica, as alterações metabólicas e as alterações de sincronia pode identificar alvos terapêuticos. Examinar o SLG basal e a duração do QRS pode personalizar as estratégias. Ensaios clínicos sobre parâmetros de estimulação, estimulação do feixe de His ou do ramo esquerdo do feixe de His podem orientar a seleção, melhorando os resultados para os pacientes.

## Conclusão

Este estudo investiga o impacto da estimulação do VD no desempenho cardíaco de pacientes com FEVE basal preservada e corações estruturalmente normais. Os achados são apresentados como associações exploratórias. A estimulação do VD foi associada a alterações adversas significativas nas funções do VE e do VD. Notavelmente, a fração de ejeção do VE e o SLG absoluto diminuíram significativamente, com reduções observadas já sete dias após a estimulação (alterações precoces) e persistindo até seis meses. Especificamente, a FEVE diminuiu 23,7 pontos percentuais absolutos em pacientes com disfunção PIVD e 41,2 pontos percentuais absolutos naqueles com PCIM, ambos definidos no acompanhamento de 6 meses.

Além disso, parâmetros da função do VD, como AF, TAPSE e velocidade da onda S, também se deterioraram ao longo do tempo. O estudo identificou variáveis-chave associadas a desfechos adversos, incluindo comprometimento precoce do SLG absoluto em sete dias, um complexo QRS pré-implante mais amplo e maiores percentuais de estimulação. Esses resultados reforçam a necessidade de identificação precoce e monitoramento rigoroso de pacientes submetidos à estimulação do VD para mitigar os riscos de PIVD) e PICM. Estratégias de manejo eficazes e abordagens de estimulação personalizadas são essenciais para melhorar os desfechos dos pacientes e reduzir as complicações relacionadas à insuficiência cardíaca.
