# Supplementary material for: Efeitos dos Marca-passos nas Funções Ventriculares e Variáveis Precoces Associadas à Cardiomiopatia Induzida
Source: Arq Bras Cardiol. 2026 Jul 31;123(7):e20250566. [Article in Portuguese] doi: 10.36660/abc.20250566 (PMC13668443; doi:10.36660/abc.20250566)
Supplement: Translation

# Translation: Effects of Pacemakers on Ventricular Functions and Early Variables Associated with Induced Cardiomyopathy

**DOI:** 10.36660/abc.20250566i

**Authors:** Sanjeev Kumar, Chandra Bhan Meena, Navida Babbar, Anil Bhandari, Dhananjay Singh Shekhawat, Ankita Meel

**Affiliations:** 1 India SMS Medical College, Jaipur – India

**Keywords:** Artificial Pacemaker, Heart Failure, Three-Dimensional Echocardiography, Cardiomyopathies

## Abstract

**Background:**

Right ventricular (RV) pacing can alter ventricular mechanics and may lead to ventricular dysfunction, even in patients with preserved baseline left ventricular ejection fraction (LVEF) and structurally normal hearts.

**Objective:**

This study examines the hemodynamic effects of right ventricular pacing on left ventricular function and volume, and identifies early variables associated with pacemaker-induced left ventricular dysfunction and cardiomyopathy in patients with dual-chamber RV pacemakers.

**Methods:**

The study enrolled patients undergoing dual-chamber RV pacemaker implantation without any structural abnormality of the heart. Data on biventricular functions, LV volumes, and global longitudinal strain (GLS) were collected with 2D, speckle tracking, and 3D echocardiography. Paired continuous variables were compared using two-sided tests with a significance level of 0.05.

**Result:**

Out of 75 patients, 20 experienced LV systolic dysfunction, 15 developed PIVD, and five developed PICM. In comparison to the control group, patients with PICM had lower GLS (17.02 vs. 19.27; p<0.05), wider QRS width (139.57 vs. 102.75; p < 0.05), and greater ventricular pacing percentage (VP%; 94.32 vs. 84.03; p = 0.06).

**Conclusion:**

RV pacing resulted in a substantial decline in LV systolic function in the short and long term. Concurrently, RV functions declined over the long term (six months). Diminished GLS at seven days, a broader pre-implantation QRS complex, and an elevation in VP% are early indicators of PIVD and PICM. Routine patient monitoring for these indicators can be beneficial for timely intervention on a case-by-case basis.

## Introduction

Patients with preserved baseline left ventricular (LV) ejection fraction (EF) and structurally normal hearts represent a growing population requiring permanent pacemaker implantation.^1-3^ Cardiac pacing, a well-established therapy for managing heart failure and arrhythmias, plays a crucial role in patients with bradycardia or atrioventricular block, where pacemakers are essential for maintaining an adequate heart rhythm.^4^ Over decades, pacing technology has evolved from single-chamber pacemakers to dual-chamber devices, and later to biventricular pacemakers in cardiac resynchronization therapy (CRT), which improves cardiac output in patients with reduced LV function.^5,6^ However, CRT is not routinely indicated in individuals with preserved EF, and its high cost and technical complexities limit its broader application.^7^

In patients requiring permanent pacemaker implantation who have preserved baseline EF and structurally normal hearts, right ventricular (RV) pacing remains the most commonly employed strategy.^8^ Prolonged RV pacing induces ventricular dyssynchrony, which disrupts normal heart function, leading to pacemaker-induced left ventricular dysfunction (PIVD) and cardiomyopathy (PICM), worsening heart failure, and increasing mortality.^9,10^ Early identification of patients at risk is crucial to prevent severe complications. ^11,12^

This study aims to investigate the hemodynamic effects of RV pacing on LV volumes and functions, assess RV function, and identify early variables associated with PIVD and PICM in patients receiving dual-chamber RV pacemakers. Furthermore, this study aims to provide insights that could guide clinical decision-making, improve patient management, and ultimately reduce the burden of heart failure in patients reliant on RV pacing.

## Patients and methodology

### Study design and approval

The study received approval from our institutional ethics committee on 04/04/2023, and all participating patients gave written informed consent. Recruitment occurred between March 2022 and March 2023, when 200 consecutive patients sought pacemaker implantation at our hospital.

### Patient selection

Patients with EF<50%, significant valvular heart disease, coronary heart disease, inadequate echo windows, persistent atrial fibrillation with delayed ventricular response, other arrhythmias potentially impacting LV function, any debilitating condition or cancer (with an anticipated survival of less than one year), prior implantation of devices, and recent cardiac surgery (within the past three months) were excluded. Of the initial pool, only 75 patients fulfilled the criteria and were thus enrolled. The study population comprised patients with preserved baseline LVEF (≥50%) and structurally normal hearts, as confirmed by echocardiography. Patients were not assessed for a clinical diagnosis of heart failure, and no heart failure classification was used as an inclusion criterion.

### Pacemaker implantation and programming

All patients in this study underwent dual-chamber RV pacemaker implantation. Implantation procedures were conducted based on the preference of the operator. In 65 patients, the RV septum (RVS) was chosen as the RV lead implantation site. The remaining ten patients underwent RV apical (RVA) pacing. The pacing site was confirmed by electrocardiogram (ECG) axis assessment post-pacing. A dual-chamber rate-modulated mode was set for all patients. The rate-responsive mode was employed to enhance patients’ quality of life and exercise capacity. Lower rate limits were set at 60 beats per minute, and upper rate limits were set at 130 beats per minute. The resting atrioventricular time delay for paced/sensed signals was set at 200/150 ms, with optimization of the dynamic atrioventricular time delay aimed at maximizing the contribution of the atrial kick to stroke volume. Strategies aimed at reducing pacing were implemented.^13^

### Data collection

The data was collected three times: before pacemaker implantation, at seven days, and six months post-pacing. The altered pacing settings were verified, and the VP% was obtained by device interrogation. During follow-up visits, evaluations included 2D and 3D echocardiography, as well as global longitudinal strain (GLS) assessment. EF% was used to classify patients with PIVD or PICM. A reduction of more than 10% while maintaining EF > 50% is defined as PIVD, while PICM is characterized by a more than 10% reduction leading to EF ≤ 50%.^14^

### Electrocardiogram

Before pacing, a standard 12-lead ECG was conducted. Data from the ECG, including heart rate, native QRS width and axis, degree of heart block, and presence of morphology similar to left bundle branch block (LBBB) or right bundle branch block (RBBB), were collected and documented. The determination of LBBB and RBBB followed established criteria. Subsequently, all ECG parameters were reassessed and documented at seven days and six months following pacing.

### 2D Echocardiography

To rule out individuals with coronary heart disease, significant valvular heart disease, or diminished EF, a complete 2D Echocardiographic examination was conducted.

### Right Ventricular Parameters

Right ventricular systolic function was assessed using fractional area change (FAC), tricuspid annular plane systolic excursion (TAPSE), and tissue Doppler-derived systolic velocity (S’) obtained from the apical four-chamber view according to current echocardiographic recommendations. Fractional area change was calculated as [(RV end-diastolic area − RV end-systolic area) / RV end-diastolic area] × 100. Tissue Doppler imaging was performed by placing the sample volume at the lateral tricuspid annulus to obtain the peak systolic (S’) velocity.

#### GLS measurement

The apical images (apical four, two, and three) were 2D gated acquired for GLS computation using industry-standard methods. To ensure clear imaging and minimize extraction, patients were instructed to stop breathing upon scanning. According to GLS calculations, the central GLS was automatically determined for each of the three apical views (Figure 1).

**Figure 1:** – Representative images for GLS measurements. GLS was calculated from three standard apical views in the 2D Echocardiographic data set

#### 3D Echocardiography

For 3D echocardiography, we used Philips Medical’s IE33 echocardiography system using an X5-1 transducer probe. A 30°x 30° pyramid exceeding 4 to 6 alternate gated cardiac cycles obtained in the left ventricle. Optimal packet size, frame rate, and comprehensive recording of the cardiovascular cycle were ensured (Figure 2).

**Figure 2:** – This figure presents two-dimensional slices from a 3D echocardiographic dataset of the left ventricular myocardium, used to calculate key cardiac parameters: EDV, ESV, SV, COP, and EF.

## Left Ventricular parameters

The program delineated the borders of the left ventricle, enabling users to make adjustments for enhanced precision as needed. LV parameters: Cardiac output (COP), Stroke volume (SV), end-diastolic volume (EDV), end-systolic volume (ESV), and EF were automatically computed once the boundaries were drawn.

## Statistical analysis

An independent data management group was tasked with maintaining and analyzing the database. To assess data distribution for continuous variables, standardized skewness and kurtosis were calculated. Normally distributed data were represented as mean ± standard deviation (SD). Categorical variables were expressed as absolute frequencies and percentages. Statistical significance was considered for all group comparisons with p < 0.05. Two-sided paired comparisons between time points were conducted using the paired Student’s t-test. Comparisons among the three LV functional outcome groups (no decline, PIVD, and PICM) were performed using one-way analysis of variance (ANOVA) for continuous variables and the χ^2^ test or Fisher’s exact test for categorical variables, as appropriate. When the overall ANOVA was statistically significant (p < 0.05), pairwise group comparisons were conducted using Tukey’s Honestly Significant Difference (HSD) post-hoc test to identify which specific groups differed significantly while controlling for the familywise error rate. Effect estimates with 95% confidence intervals were calculated for key functional parameters.

## Results

### Baseline characteristics and risk factors for study participants

A total of 75 patients were included and completed the defined observations 6 months after subsequent testing. The average patient was 65, between the ages of 12 and 97. Twenty patients (26.7%) had diabetes, and 32 patients (42.7%) had hypertension. In addition, 22 patients (29.3%) had pre-existing LBBB or RBBB (Table 1). Echocardiography confirmed a structurally normal heart in each patient. A central illustration is provided summarizing the study design, timeline of pacemaker-related changes, and key variables associated with ventricular function outcomes in patients with dual-chamber RV pacemakers.

**Table 1:** – Summary of patients’ characteristics

Factors	Number	%
**Age (Mean ± SD)**	65.30 ± 10.09	
**Sex**		
Male	40	53.33
Female	35	46.67
**DM**	20	26.66
**HTN**	32	42.67

DM: diabetes mellitus; HTN: hypertension.

### Ventricular response to pacing

We observed a significant decline in LV volumes and functions after pacing (Table 2). Specifically, EDV and SV exhibited a noticeable reduction as early as seven days post-pacing. Additionally, ESV increased at the six-month mark following pacing compared to baseline values. EF and GLS demonstrated a marked decline at the seven-day point, with these decreases continuing progressively through to the six-month post-pacing period, compared to the baseline measurements.

**Table 2:** – Comparison of different parameters in dual-chamber pacing groups, including all the measured parameters at the three time intervals (pre-pacing, at 1 week, and 6 months post-pacing)

Parameters	Pre-pacing	Post- pacing at day 7	Post- pacing at 6 months	P-value (Baseline vs 6 months)	Mean change (Baseline to 6 months; 95% CI)
EDV (ml)	108.25 ± 12.49	91.65 ± 7.93*	94.26 ± 12.36^$^	<0.001	–14.0 (IC 95% –16.5 a –11.6)
ESV (ml)	34.38 ± 6.43	34.92 ± 6.18	45.32 ± 6.20^$#^	<0.001	+10.9 (IC 95% +10.5 a +11.3)
SV (ml)	72.28 ± 10.20	58.48 ± 8.32*	53.01 ± 5.11^$#^	<0.001	–19.3 (IC 95% –21.1 a –17.5)
HR (bpm)	42.44 ± 5.90	75.75 ± 5.69*	73.89 ± 4.86^$^	<0.001	+31.5 (IC 95% +29.8 a +33.2)
COP (L/m)	3.02 ± 0.52	4.42 ± 0.52*	3.90 ± 0.52^$#^	<0.001	+0.88 (IC 95% +0.80 a +0.96)
EF (%)	66.84 ± 12.19	58.84 ± 17.18*	52.04 ± 14.18^$#^	<0.001	–14.80 (–19.07 a –10.53)
Absolute GLS (%)	19.54 ± 1.98	15.22 ± 1.97*	14.33 ± 1.93^$^	<0.001	–5.20 (IC 95% –5.84 a –4.58)

Values are presented as mean ± standard deviation (SD). P-values represent paired Student’s t-test comparisons between baseline and six-month measurements. *p < 0.001 vs baseline (baseline vs day 7); $p < 0.001 vs baseline (baseline vs 6 months); #p < 0.001 vs day 7 (day 7 vs 6 months). EDV: end-diastolic volume; ESV: end-systolic volume; SV: stroke volume; COP: cardiac output; EF: ejection fraction; GLS: global longitudinal strain

RV functions, FAC, TAPSE, and S’ velocity were initially maintained seven days post-pacing. However, these parameters showed a significant decline by the six months after pacing compared to their respective baseline values (Table 3). This data highlights the impacts of pacing on both LV and RV functions over time.

**Table 3:** – Comparison of different parameters in dual-chamber pacing groups, including all the measured parameters of the right ventricle at the three time intervals (pre-pacing, at 1 week, and 6 months post-pacing)

Parameters	Pre-pacing	Post- pacing at day 7	Post- pacing at 6 months	P-value (Baseline vs 6 months)	Mean change (Baseline to 6 months; 95% CI)
FAC (%)	42.28 ±2.16	41.82 ± 2.39	39.55 ± 3.28^$#^	<0.001	–1.95 (–3.85 a –0.06)
TAPSE (mm)	18.39 ± 2.20	17.77 ± 2.31	15.91 ± 2.98^$#^	<0.001	–3.21 (–5.13 a –1.30)
S’ Velocity (cm/sec)	13.43 ± 1.03	13.51 ± 1.30	10.61 ± 1.44^$#^	<0.001	–2.38 (–3.23 a –1.53)

Values are presented as mean ± standard deviation (SD). P-values represent paired Student’s t-test comparisons between baseline and six-month measurements. *p < 0.001 vs baseline (baseline vs day 7); $p < 0.001 vs baseline (baseline vs 6 months); ^#^p < 0.001 vs day 7 (day 7 vs 6 months). FAC: fractional area; TAPSE: tricuspid annular plane systolic excursion.

### Risk stratification for pacing-induced cardiomyopathy

At seven days post-pacing, 55 patients had less than 10% reduction in EF (termed as the control group), while 20 had a decline in EF≥10% compared to pre-implantation EF (Table 4). A subgroup analysis of these 20 patients identified 15 patients with PIVD (EF≥50%) and five patients with PICM (EF<50%). At six months, the mean EF% in the PIVD and PICM groups fell by 23.7% and 41.2%, respectively.

**Table 4:** – Comparison of risk factors in study groups: (RVS- RV septal pacing, RVA- RV apical pacing)

Predisposing Factor	No decline in LVEF (55)	Decline in LVEF		p- value
		PIVD (15)	PICM (5)	
**Age**	65.30 ± 10.09	57.6 ± 11.72	66.4 ± 11.85	0.03
**Sex**	n (%)	n (%)	n (%)	0.81
Male	30 (54.55)	7 (46.67)	3 (60)	
Female	25 (45.55)	8 (53.33)	2 (40)	
**DM**	14 (25.45)	3 (20)	3 (60)	0.06
**HTN**	24 (43.63)	5 (33.33)	3 (60)	0.07
**Lead site**				0.67
RVA (10)	7 (12.7)	2 (13.33)	1 (20)	
RVS (65)	48 (87.27)	13 (87.67)	4 (80)	
**Pre-existing BBB**				0.06
No BBB	44 (80)	9 (60)	0 (0.0)	
RBBB	8 (14.55)	4 (26.67)	4 (80)	
LBBB	3 (5.45)	2 (13.33)	1 (20)	
**QRS Axis**				0.78
N	38 (69.1)	11 (73.4)	1 (20)	
Left	5 (9.1)	2 (13.3)	2 (40)	
Right	12 (21.8)	2 (13.3)	2 (40)	
**Native pre-implant QRS width (ms)**				
Pre-pacing	102.75 ± 15.37	115.61 ± 19.48	139.57 ± 19.77	<0.05
Post-pacing	147.24 ± 13.25	153.11 ± 12.83	157.57 ± 17.35	0.74
**VP%**				
7 days	84.03 ± 14.64	82.53 ± 9.67	94.32 ± 5.1	0.06
6 months	87.68 ± 11.34	89.79 ± 8.79	100.00 ± 0.0	0.021
**Pacing threshold**				
7 days	0.56 ± 0.11	0.54 ± 0.11	0.49 ± 0.1	0.37
6 months	0.84 ± 0.15	0.87 ± 0.12	0.82 ± 0.32	0.92

PIVD: Pacemaker-Induced Ventricular Dysfunction; PICM: Pacemaker-Induced Cardiomyopathy LVEF: left ventricular ejection fraction; RVA: right ventricular apical; RVS: right ventricular septum; LBBB: left bundle branch block; RBBB: right bundle branch block; VP%: ventricular pacing percentage.

The PIVD and PICM groups showed a decline in GLS at seven days post-pacing compared to pre-implantation GLS (Figure 3a) and had wider pre-implantation QRS widths than the control group (Figure 3b). The PICM group also had a low pre-implantation GLS (Figure 3a) and a higher VP% (Table 4) compared to both PIVD and the control group. Where one-way ANOVA was statistically significant (p < 0.05), Tukey’s Honestly Significant Difference (HSD) post-hoc test was applied to identify specific between-group differences; complete pairwise results are presented in Table 5. Pre-implantation QRS width differed significantly across the three groups (p < 0.05), with Tukey’s HSD confirming a stepwise increase across all pairwise comparisons: No Decline vs. PIVD (p = 0.025), No Decline vs. PICM (p < 0.001), and PIVD vs. PICM (p = 0.017). VP% at six months differed significantly across groups (p = 0.021), driven primarily by a higher VP% in the PICM group compared to No Decline (p = 0.039). Age showed a significant overall difference (p = 0.030), with a younger mean age in the PIVD group compared to No Decline (p = 0.038). For all remaining variables, the omnibus ANOVA was non-significant (p ≥ 0.05) and post-hoc testing was therefore not performed (Table 5).

**Figure 3:** – Early variables associated with PIVD and PICM (a) A wider pre-implantation QRS complex and (b) Low post-implantation GLS suggest a higher risk of cardiac dysfunction.

**Table 5:** – Tukey’s Honestly Significant Difference (HSD) Post-hoc Pairwise Comparisons Following One-Way ANOVA for Risk Stratification Variables (Table 4)

Variable	No Decline vs PIVD	No Decline vs PICM	PIVD vs PICM
Age	p = 0.038*	p = 0.973 (ns)	p = 0.245 (ns)
Native Pre-implant QRS Width (ms)	p = 0.025*	p < 0.001***	p = 0.017*
VP% at 6 months (%)	p = 0.772 (ns)	p = 0.039*	p = 0.154 (ns)

Variables with non-significant ANOVA (p ≥ 0.05): post-hoc not applicable. ND: No Decline; PIVD: Pacemaker-Induced Ventricular Dysfunction; PICM: Pacemaker-Induced Cardiomyopathy; ns: not significant; N/A, not applicable (post-hoc testing not performed when omnibus ANOVA p ≥ 0.05). Significance: * p < 0.05; ** p < 0.01; *** p < 0.001.

## Discussion

In this exploratory study, pacing-induced LV systolic dysfunction was more prevalent, manifesting within six months post-implantation. A wider pre-implantation QRS complex, lower post-implantation GLS(an early change observed at seven days), and a greater pacing percentage were associated with PICM and PIVD development.

We observed both short-term and long-term adverse impacts on LV volume and functions. Within seven days, SV was significantly reduced in association with a drop in EDV. This may be related to pacing at higher heart rates, shortening the cardiac cycle duration, particularly diastole, and reducing the time for ventricular filling. This decrease in EDV reduces the myocardial stretch, consistent with a drop in SV (Frank-Starling law).^16^ Yu et al. investigated the acute effects of RVA in 35 patients with sick sinus syndrome, observing a decline in LV EDV and EF.^17^ Reduced EF may be associated with pacing-induced remodelling of the LV structure, which was observed to alter LV volumes, mainly increasing ESV.^18^ At the second follow-up, we also observed a further drop in EF and increased ESV, which may be related to the pacing-induced remodelling of the LV structure.

We identified 20 (27%) patients with ≥10% reduction in EF, further categorized into patients with PIVD (20%) or PICM (7%). It is important to note that the number of PICM cases was small (n=5), which limits definitive conclusions and precludes multivariable adjustment. The incidence of PICM in our study aligned with previous reports of 5-27%, with the variation being dependent on the PICM defining criteria and the length of follow-up.^14^ Lee et al. found a 20.5% incidence of PICM after an average follow-up duration of 15.6 years, while Dawood et al. reported an incidence rate of 12% with a six-month follow-up.^19,20^

A lower absolute pre-implantation GLS was observed in patients who subsequently developed PICM. A significant decline in absolute GLS was noted at seven days in patients with either PIVD or PICM. An increasing body of research highlights the negative association between pacing and myocardial contraction. Delgado et al. reported the effects of RVA pacing in 25 individuals with structurally normal hearts, finding greater dyssynchronous LV contraction and impaired LV longitudinal shortening and twisting during RVA pacing.^21^ Babu et al. followed 36 patients with EF above 45% for six months after permanent pacemaker implantation, noting a significant reduction in GLS in 63.9% of patients by six months, with a notable reduction in overall longitudinal strain in seven patients 24 hours post-implantation.^22^ Recent studies indicate that patients with lower GLS may be at risk for PICM, identifying lower LV peak GLS as an independent indicator of LV dyssynchrony.^23^

The PICM group had a wider pre-implantation QRS complex and a higher VP% at both follow-ups. Another study found male gender, lower baseline EF, and a wide native QRS complex were variables associated with PICM.^24^ Kim et al. also linked PICM with abnormal post-pacing QRS axis, which our study could not corroborate due to the small sample size.^25^

We found a significant reduction in TAPSE, S’ velocity, and FAC at the second follow-up, indicating RV dysfunction. Sinkar et al. assessed RV dimensions and function in 60 patients six months after pacemaker implantation, finding a decline in RV function parameters.^26^ Porapakkham et al. analyzed factors affecting RV dysfunction in 96 patients, noting that factors affecting LV function, such as pacing lead site, pacing mode, and VP%, did not significantly impact RV function.^27^

Our study was limited owing to the small sample size along with a short follow-up period, although it was statistically significant. An extended follow-up would allow one to predict a greater incidence of pacing-induced malfunction. Further, 87% of patients in our study underwent RVS pacing, with only 10 (13%) patients having RVA pacing. Due to this, our study is limited in analysing the role of the RV lead site on ventricular functions and cardiomyopathy. Some studies have shown that RVS pacing has better clinical outcomes over the short and long term. Karpawich et al. compared apical and mid-septal pacing sites in canines, finding that septal pacing prevented remodelling over a four-month follow-up period.^28^ They found preserved mitochondrial morphology, no calcification, or degenerative changes in the septal-paced group.

Tse et al. reported that patients with RVA pacing had significantly broader average QRS duration, lower EF, and a higher incidence of myocardial perfusion defects and regional abnormalities than patients with RVS pacing.^29^ They further concluded that preserving synchronous ventricular activation during RVS pacing mitigates the long-term detrimental effects of RVA pacing on myocardial perfusion and function.^16^ However, Ng et al. demonstrated in a long-term study that septal pacing resulted in worse LV dyssynchrony and a more impaired circumferential strain than apical pacing.^30^

Several potential sources of bias should be considered when interpreting the findings. Strain-based and right ventricular functional measurements are inherently operator-dependent and influenced by image quality and tracking algorithms. Although standardized acquisition protocols and experienced operators were used, inter-observer variability cannot be completely excluded and may have affected the precision of strain and RV functional indices. Despite its novel contributions, the study has a few limitations. Although no a priori sample size calculation was performed, a post-hoc power analysis based on the observed EF reduction of 14.8 percentage points (SD ~13.0) in 75 patients demonstrated >99% power at α = 0.05, and the observed GLS reduction of 5.2% (SD ~2.0) similarly showed >99% power. These findings suggest adequate statistical power for the primary echocardiographic outcomes; however, the small number of PICM cases limited subgroup analyses and multivariable modelling. Due to the low PICM event count, multivariable adjustment was not possible, and residual confounding cannot be excluded. Strain and 3D echocardiographic measurements are operator-dependent and susceptible to image-quality variation.

Further studies with extended follow-up are needed to elucidate the long-term effects of pacing on LV function, as longer observation may reveal more incidences of dysfunction. Larger sample sizes will enhance statistical power for definitive conclusions and subgroup analyses, particularly regarding pacing sites. Given differential outcomes of RVA and RVS pacing, comparative studies with standardized assessments of dyssynchrony and perfusion are essential. Investigating myocardial fibrosis, metabolic changes, and synchrony alterations may identify therapeutic targets. Examining baseline GLS and QRS duration can personalize strategies. Trials on pacing parameters, His-bundle, or left-bundle branch pacing could guide selection, improving patient outcomes.

## Conclusion

This study investigates the impact of RV pacing on patient cardiac performance in patients with preserved baseline LVEF and structurally normal hearts. These findings are presented as exploratory associations. RV pacing was associated with significant adverse changes in both LV and RV functions. Notably, LV ejection fraction and absolute GLS declined significantly, with reductions observed as early as seven days post-pacing (early changes) and continuing through six months. Specifically, EF decreased by 23.7 absolute percentage points in patients with PIVD and 41.2 absolute percentage points in those with PICM, both defined at 6-month follow-up.

Additionally, RV function parameters such as FAC, TAPSE, and S’s velocity also deteriorated over time. The study identified key variables associated with adverse outcomes, including early impairment of absolute GLS at seven days, a wider pre-implantation QRS complex, and higher pacing percentages. These results underscore the need for early identification and close monitoring of patients undergoing RV pacing to mitigate risks of PIVD and PICM. Effective management strategies and tailored pacing approaches are essential to improve patient outcomes and reduce heart failure-related complications.
